# Supplementary material for: Zika virus noncoding RNA suppresses apoptosis and is required for virus transmission by mosquitoes
Source: Nat Commun. 2020 May 5;11:2205. doi: 10.1038/s41467-020-16086-y (PMC7200751; doi:10.1038/s41467-020-16086-y)
Supplement: Supplementary file 1 — Supplementary Information [file 41467_2020_16086_MOESM1_ESM.pdf]

Supplementary information

Zika virus noncoding RNA suppresses apoptosis and is required for virus transmission by mosquitoes

Slonchak et al.

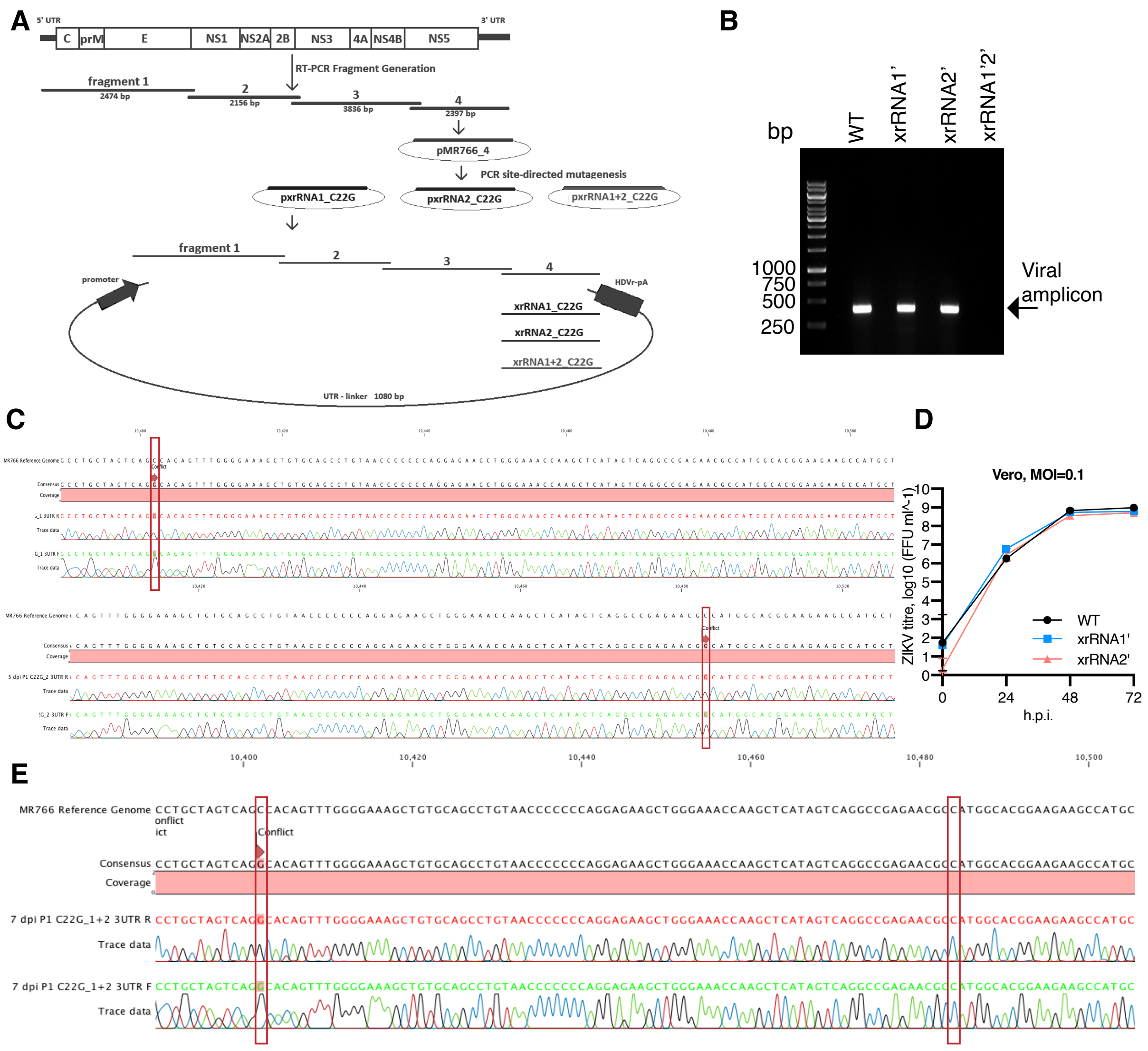

Supplementary figure 1. Generation and analysis of Zika MR766 mutants containing C to G substitution in XRN-1 resistant structures of 3'UTR. (A) Schematic overview of the circular polymerase extension reaction (CPER)-based approach used for generation of the ZIKV mutants. Overlapping fragments of ZIKV cDNA spanning the entire viral genome were PCR amplified using proofreading polymerase. Fragment 4, containing the 3'UTR was cloned in pUC19 vector and subjected to PCR-directed mutagenesis. Plasmids containing mutated fragments were then used to PCR-amplify the variants of fragment 4 containing mutations. Equimolar amounts of fragments 1-3, WT or mutated fragment 4, and DNA linker fragment, containing CMV (for mammalian system) or OpIE2 (for insect system) promoter and HDV ribozyme were mixed in equimolar amounts and subjected to CPER assembly. The resulted infectious circular cDNAs were transfected into Vero 76 or C6/36 cells. (B) Agarose gel electrophoresis showing the RT-PCR amplicons obtained from culture fluids of Vero 76 cells transfected with CPER-generated infectious cDNA. Viral RNA was isolated from culture fluids, treated with DNase I and subjected to RT-PCR with primers specific to ZIKV 3'UTR. PCR-products were gel-purified and sequence using the Sanger method. Panel shows a representative image from two independent experiments that produced similar results. (C) Sanger sequencing of ZIKV mutants produced in Vero cells (passage 0), confirming incorporation of the correct mutations (conflict sign) into xrRNA structures of viral 3'UTRs. (D) Growth kinetics of WT ZIKV and mutants deficient in individual sfRNAs in Vero 76 cells. Cells were infected with each virus at MOI=0.1. Culture fluids were harvested from the infected cells at the indicated time points and viral titres were determined by IPA on C6/36 cells. Values are the means of 3 biological replicates with standard deviations. (E) Sanger sequencing of secreted xrRNA1'2' mutant virus produced in C6/36 cells, showing the correct mutation in xrRNA1 and reversion of the mutation in xrRNA2 to the wild type sequence.

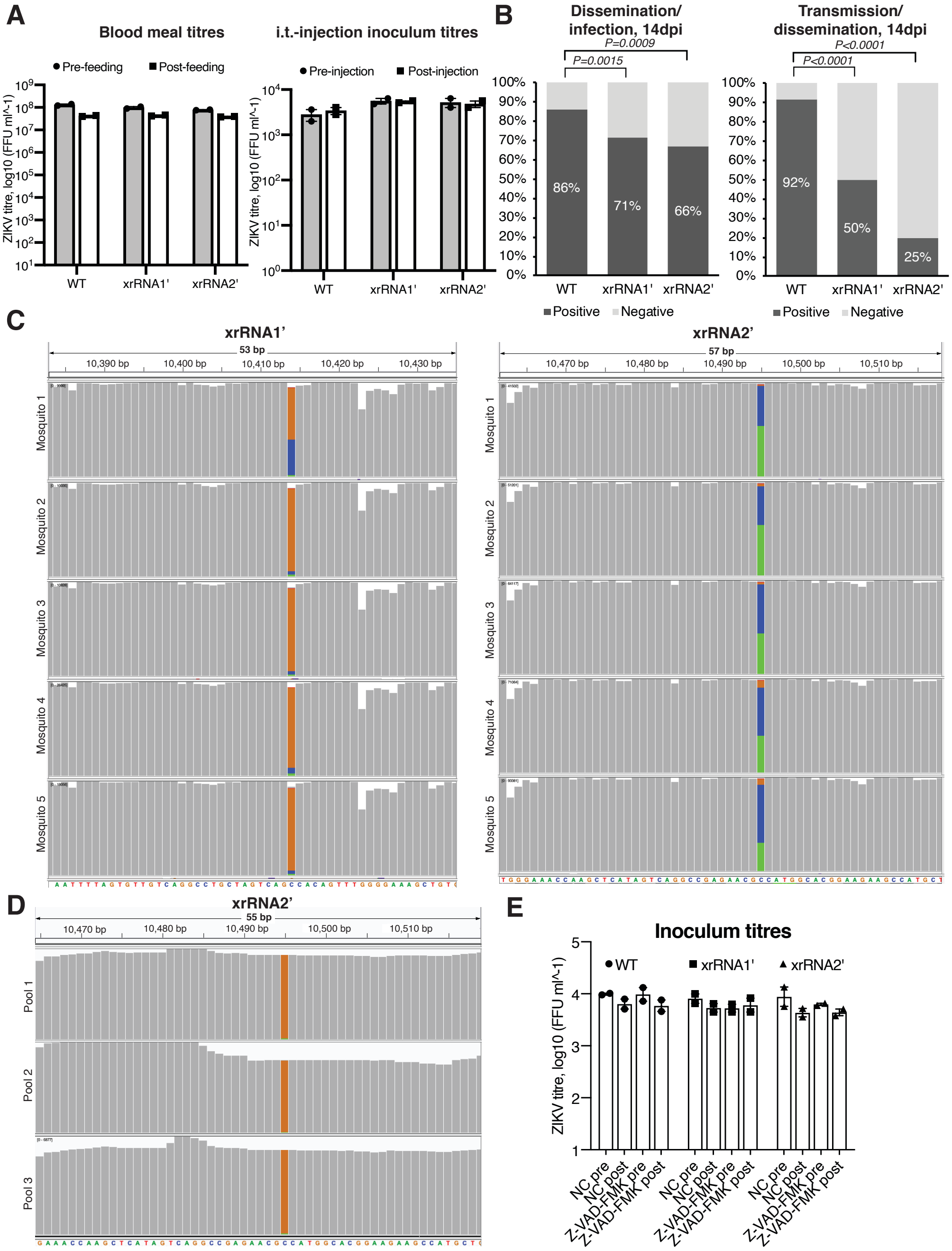

Supplementary figure 2. Inoculums used for *in vivo* experiments and analyses of viral diversity in the mosquitoes. (A) ZIKV titres in the inoculums used for mosquito infection. Pre- and post-exposure viral titres in the infectious blood meals or inoculum were determined by immuno-plaque assay. (B) Dissemination to infection and transmission to dissemination ratios in mosquitoes inoculated via blood feeding. The infection, dissemination and transmission rates were determined as percentage of ZIKV-positive bodies, legs and wings, and saliva samples respectively. Statistical analysis is by independent two-sided chi-squared tests. Sample sizes are the same as in Figure 2D (C) Nucleotide frequencies in each position of xrRNA1 and xrRNA2 in viral populations present in mosquitoes at 14 days after exposure to blood meals containing xrRNA1' and xrRNA2' mutants respectively. Viral RNA was isolated from the homogenates of the individual ZIKV-positive mosquito bodies, viral 3'UTRs were amplified by RT-PCR, amplicons were purified and sequence using the Oxford Nanopore MinION platform. Reads that passed quality control were adapter trimmed and mapped to viral genome. (D) Nucleotide frequencies in each position of xrRNA2 in viral populations present in mosquitoes at 10 days after intrathoracic injection with the inoculum containing xrRNA2' ZIKV mutant. Total RNA was isolated from three pools of 10 mosquitoes in each and used for preparation of rRNA-depleted libraries. Sequencing was performed on the Illumina HiSeq platform and reads were mapped to viral genome. In C-D, orange shading indicates for C->G substitution in the position of interest, blue corresponds to C (WT genotype), green is A and red is U. (E) ZIKV titres in viral inoculums before (pre-) and after (post-) inoculation of mosquitoes for the experiment with the caspase inhibitor. Mosquitoes were i.t. injected with 200nl of inocula containing 10^4 FFU/ml of each virus either with 625uM of pan-caspase inhibitor Z-VAD-FMK or with a vehicle control (NC). Values in (A-B) and (E) are the means from two independent inoculations +/- SEM.

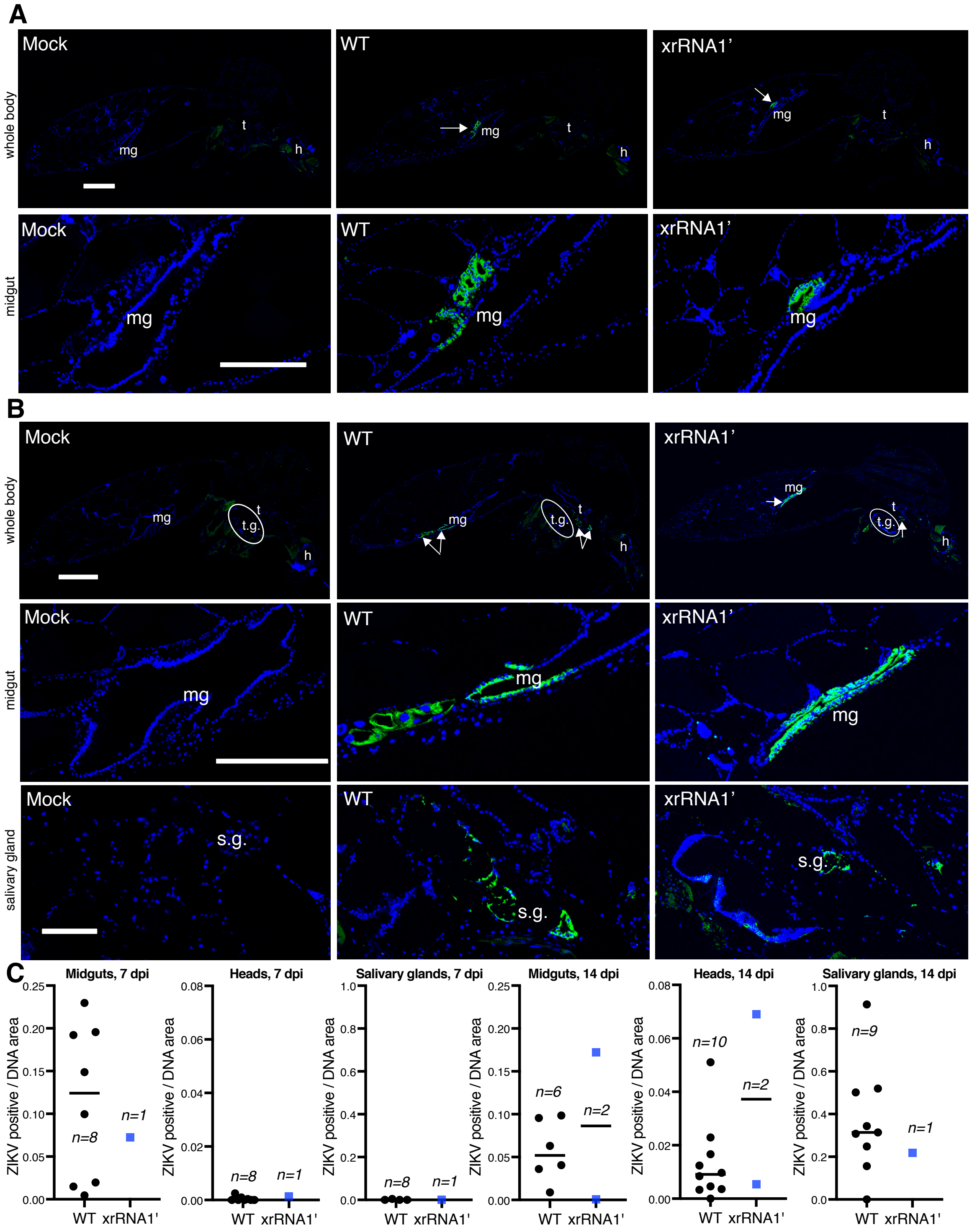

Supplementary figure 3. **Immunohistological detection of ZIKV infection in tissues of mosquitoes exposed to an infectious blood meal.** (A) ZIKV infection in mosquito tissues at 7 days after feeding. Bottom panels show magnified regions of the midguts with the sites of infection. (B) ZIKV infection in mosquito tissues at 14 days after feeding. Bottom panels show magnified midguts and salivary glands of the respective mosquitoes. Arrows in (A-B) point to the sites of infection on low magnification images; mg – midgut, t – thorax, h – head, s.g. – salivary glands, t.g. – thoracic ganglia. ZIKV detection was performed by immunofluorescent staining for NS1 protein (green); blue pseudo colour is a counterstaining for nuclear DNA (DAPI). Images are representative microphotographs of 6-10 individual mosquitoes per group that showed similar results. Scale bars are 500 µm for whole body, 250 µm for midgut, 100 µm for salivary gland, and apply to all images in each row. (C) Quantification of ZIKV-infected areas in organs and tissues of mosquitoes. Graphs show individual and median (horizontal line) values; sample sizes values (n) indicate the numbers of biologically independent mosquitoes.

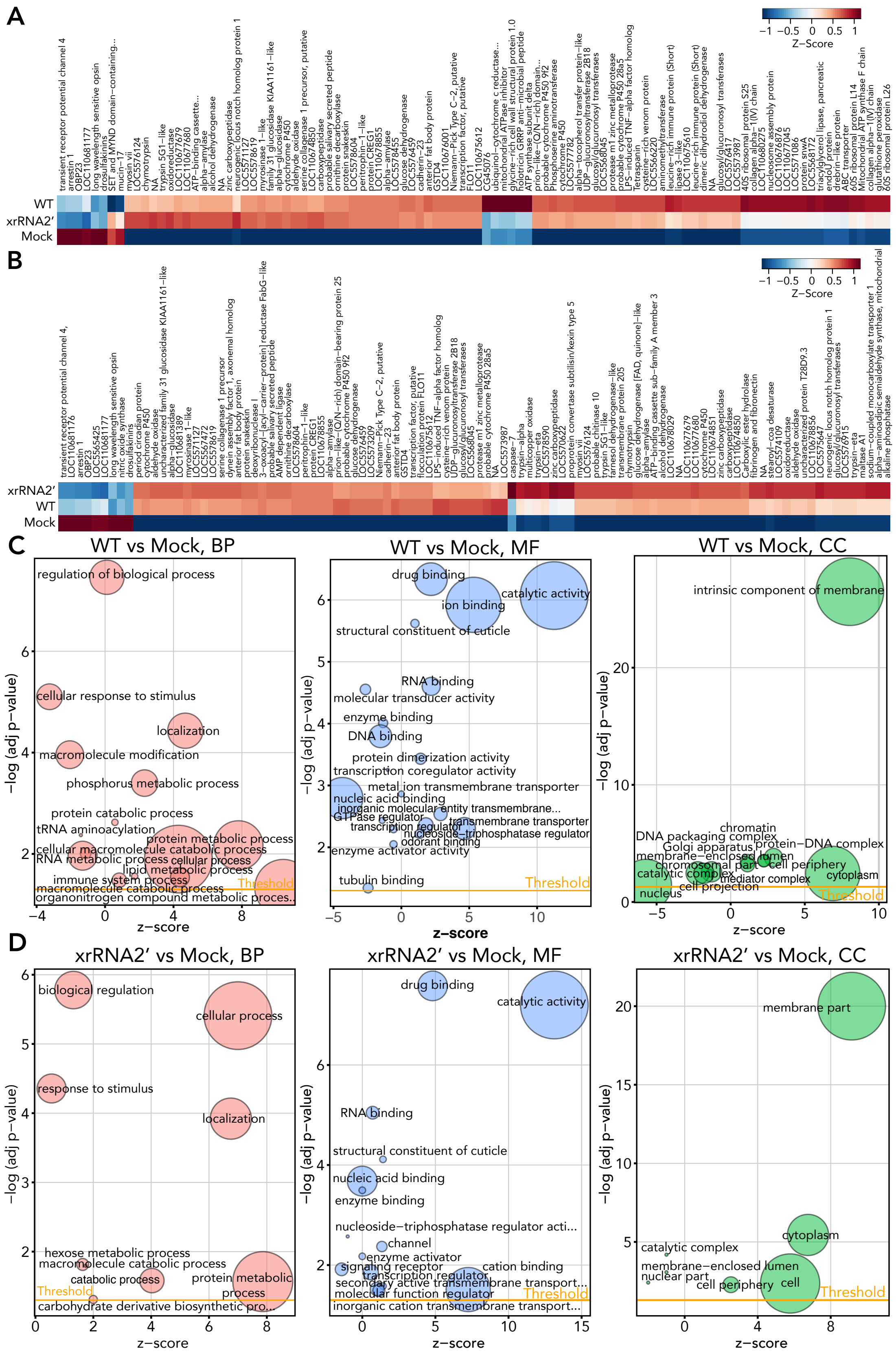

Supplementary figure 4. Differential gene expression in mosquitoes infected with WT and xrRNA2' ZIKV mutant compared to mock infected mosquitoes. (A,B) Top 100 most differentially expressed genes in mosquitoes infected with WT virus (A) and xrRNA2' ZIKV mutant virus (B). (C,D) Enriched gene ontology terms associated with differentially expressed genes in mosquitoes infected with WT(C) and the xrRNA2' ZIKV mutant (D). The size of bubbles reflects the number of DEGs associated with each GO term. Z-scores indicate the direction of change in overall expression of the genes related to the GO categories – positive z-score suggests up-regulation and negative – down-regulation. BP – biological processes, MF – molecular functions, CC – cellular components; adj P-Value – P-values were adjusted for type II error using Bonferroni correction.

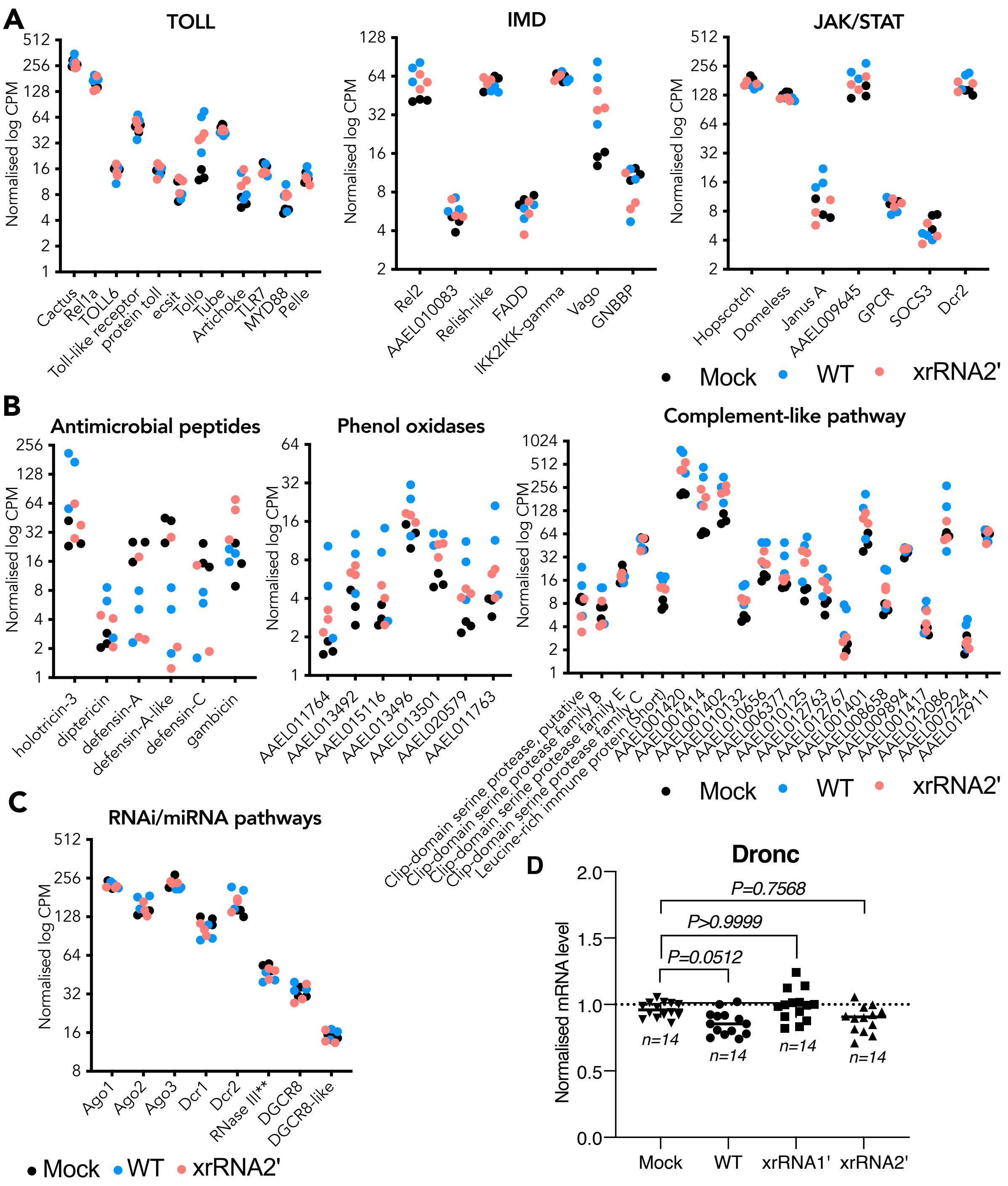

Supplementary figure 5. Expression of innate immune genes in mosquitoes infected with WT and xrRNA2' ZIKV. (A) Expression of the components of Toll, IMD and Jak-STAT pathways. (B) Expression of the effector genes of humoral immune response. (C) Expression of genes involved in RNAi/miRNA pathways. Gene expression in (A-C) was determined by RNA-Seq and individual values from three biological replicates are shown. Data are normalised to library sizes and composition bias. For statistical significance refer to tables S1-3. (D) Expression of caspase Dronc in individual mosquitoes at 10 days after i.t. injection with WT, xrRNA1' and xrRNA2' viruses as measured by qRT-PCR. Expression values were determined using the $\Delta\Delta C_T$ method with normalisation to mRNA level of *PRL11* housekeeping gene. Graph shows individual and median values. Sample sizes (n) are indicated for each group. For the levels of viral genomic RNA in the samples refer to Fig 4D. Statistical analysis was performed using the two-sided Kruskal-Wallis test with Dunn's correction for multiple comparisons.

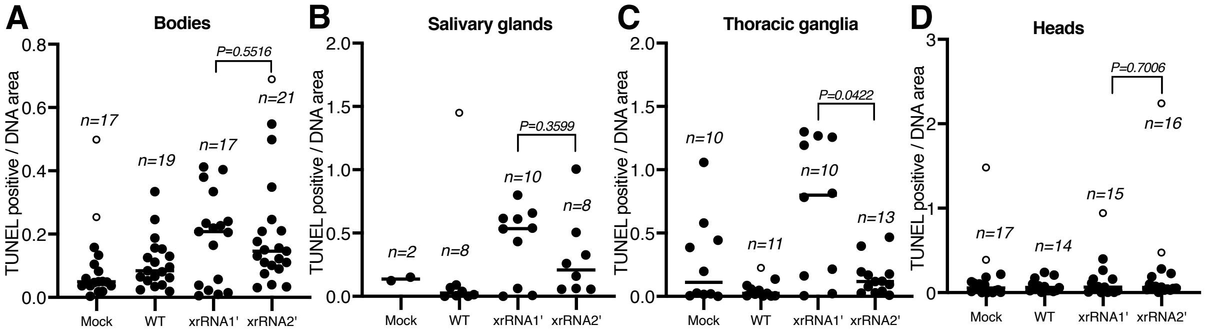

Supplementary figure 6. Statistical comparison of apoptosis rate in the tissues of mosquitoes inoculated with xrRNA1' and xrRNA2' mutants. Histological sections were prepared from mosquitoes inoculated via intrathoracic injection at 10 dpe and subjected to TUNEL staining. DAPI counterstaining of nuclear DNA was used to normalize the intensity of TUNEL signal. Outliers (shown as open circles) were identified using ROUT method with Q=1% (medium stringency) and cleaned data was analysed by two-sided Mann-Whitney U-test, no multiple comparisons were performed. Graphs show individual and median values in each group. Sample sizes are indicated on the graph.

Supplementary table 1. DNA oligonucleotides used in the study

**Name**	**Sequence 5' -> 3"**
**CPER fragment amplification primers**	
MR766UTRlinker_F	GTGTGGGGAAATCCATGGTTTCTGGGTCGGCATGGCATCTCCACC
MR766UTRlinker_R	GTCTGACTCACACAGATCAACAACTCGGTTCACTAAACGAGCTCTGCTTATATAG
MR766_1F	AGTTGTTGATCTGTGTGACTCAGAC
MR766_1R	CAGAAACAGCCGTGGAGAGGAAG
MR766_2F	CTTCCTCTCCACGGCTGTTTCTG
MR766_2R	CGCCACTCCTTTTCCCAGTCTTC
MR766_3F	GAAGACTGGGAAAAGGAGTGGCG
MR766_3R	CCTCCTCATATTTCACTGGCCTCC
MR766_4F	GGAGGCCAGTGAAATATGAGGAGGATGTGAACCTCGGCT
MR766_4R	AGAAACCATGGATTTCCCCACACCGGCCGC
**Mutagenesis primers**	
xrRNA1_C22G_F	TGCTAGTCAGGCACAGTTTGG
xrRNA1_C22G_R	GGCCTGACAACACTAAAATTGG
xrRNA2_C22G_F	GCCGAGAACGGCATGGCACGG
xrRNA2_C22G_R	CTGACTATGAGCTTGGTTTCCCAGC
**Sanger sequencing primers**	
MR766_3UTR_Seq_F	GGTCCACACCCGGAGTGTTGT
MR766_3UTR_Seq_R	CCAGCGTGGTGGAAACTCT
**Northern blotting probe**	
Zika sfRNA probe	AGAAACCATGGATTTCCCCACA
**qRT-PCR primers**	
Caspase7_F	GGAAGAGTGAAAGAGAGGGATTG
Caspase7_R	CCTTACCTATCCACAGCCTACTA
ZIKV_F	CGGAGACCCTAGAGAGACCATTAT
ZIKV_R	CATCTTCCCGATGCCCTTATT
RPL11_F	GGGTCTGGACTTCTACGTTGTGCT
RPL11_R	CCAATTCATGGGGTCTTCCTTGG
