# Supplementary material for: Zika virus noncoding RNA suppresses apoptosis and is required for virus transmission by mosquitoes
Source: Nat Commun. 2020 May 5;11:2205. doi: 10.1038/s41467-020-16086-y (PMC7200751; doi:10.1038/s41467-020-16086-y)
Supplement: Supplementary file 3 — Reporting Summary [file 41467_2020_16086_MOESM3_ESM.pdf]

nature research

Corresponding author(s): Alexander Khromykh

Last updated by author(s): Apr 3, 2020

Reporting Summary

Nature Research wishes to improve the reproducibility of the work that we publish. This form provides structure for consistency and transparency in reporting. For further information on Nature Research policies, see Authors & Referees and the Editorial Policy Checklist .

Statistics

For all statistical analyses, confirm that the following items are present in the figure legend, table legend, main text, or Methods section.

n/a	Confirmed	
☐	☒	The exact sample size (*n*) for each experimental group/condition, given as a discrete number and unit of measurement
☐	☒	A statement on whether measurements were taken from distinct samples or whether the same sample was measured repeatedly
☐	☒	The statistical test(s) used AND whether they are one- or two-sided<br>*Only common tests should be described solely by name; describe more complex techniques in the Methods section.*
☒	☐	A description of all covariates tested
☐	☒	A description of any assumptions or corrections, such as tests of normality and adjustment for multiple comparisons
☐	☒	A full description of the statistical parameters including central tendency (e.g. means) or other basic estimates (e.g. regression coefficient) AND variation (e.g. standard deviation) or associated estimates of uncertainty (e.g. confidence intervals)
☐	☒	For null hypothesis testing, the test statistic (e.g. *F*, *t*, *r*) with confidence intervals, effect sizes, degrees of freedom and *P* value noted<br>*Give P values as exact values whenever suitable.*
☒	☐	For Bayesian analysis, information on the choice of priors and Markov chain Monte Carlo settings
☒	☐	For hierarchical and complex designs, identification of the appropriate level for tests and full reporting of outcomes
☒	☐	Estimates of effect sizes (e.g. Cohen's *d*, Pearson's *r*), indicating how they were calculated

Our web collection on statistics for biologists contains articles on many of the points above.

Software and code

Policy information about availability of computer code

Data collection	N/A
Data analysis	For Illumina sequencing Quality control of raw sequencing data was performed using FastQC software v.0.72. Data was then trimmed to remove PCR primers, adapters and short reads using TRIMMOMATIC v.0.36.4 with the following settings: ILLUMINACLIP: TruSeq3-PE:2:30:10 LEADING:32 TRAILING:32 SLIDINGWINDOW:4:20 MINLEN:25 and subjected to another quality analysis with FastQC. Trimmed reads were mapped to the Ae. aegypti genome v.5.1 45 using HISAT2 v.2.1.0 allowing 2 mismatches. Feature count was performed using HTSEQ v.0.9.1.<br>For Nanopore sequencing base calling and demultiplexing was performed using Oxford Nanopore MinKNOW software (release 10/05/2018). Adapter sequences were then removed, and reads were quality-trimmed using Porechop v.0.2.3 (OmicX, France). Reads were mapped to the reference sequence of ZIKVMR766 genome using Bowtie2 v.2.3.4.3, mapping was visualised and percentage of nucleotides in the positions of interest were determined using Integrative Genomics Viewer v.2.8.1 (Broad Institute, USA).<br>Differential gene expression analysis was performed using edgeR v.3.24.0. Gene expression data were plotted using ggplot2 v.3.1.0 and gplots v.3.0.1 with colour pallets generated by RColorBrewer v.1.1-2. Gene ontology analysis was performed using the R-package TopGO v.2.34.0 and GO annotations obtained from VectorBase. Fisher's Exact Test was applied. GO enrichment data was then combined with expression data, z-scores were calculated and results were plotted using the R package GOplot v.1.0.246. Protein-protein interactions were identified using STRING v11.0 analysis software.

For manuscripts utilizing custom algorithms or software that are central to the research but not yet described in published literature, software must be made available to editors/reviewers. We strongly encourage code deposition in a community repository (e.g. GitHub). See the Nature Research guidelines for submitting code & software for further information.

Data

Policy information about availability of data

All manuscripts must include a data availability statement. This statement should provide the following information, where applicable:

- Accession codes, unique identifiers, or web links for publicly available datasets
- A list of figures that have associated raw data
- A description of any restrictions on data availability

All data is available within the article, supplementary information or source data file. RNA-Seq data generated in this study are available in Gene Expression Omnibus database [https://www.ncbi.nlm.nih.gov/geo/query/acc.cgi?acc=GSE131827] with accession number GSE131827. Raw data underlaying the results shown in figures 1B-C, 2A-H, 3C, 4D, 5B,C, 6A-C, supplementary figures 1B,D, 2A,B,E, 3C, 5D and 6A-D are provided in Source Data file.

Field-specific reporting

Please select the one below that is the best fit for your research. If you are not sure, read the appropriate sections before making your selection.

☒ Life sciences ☐ Behavioural & social sciences ☐ Ecological, evolutionary & environmental sciences

For a reference copy of the document with all sections, see nature.com/documents/nr-reporting-summary-flat.pdf

Life sciences study design

All studies must disclose on these points even when the disclosure is negative.

Sample size	Sample size was estimated using G*Power package
Data exclusions	No data was excluded
Replication	All experiments were repeated at least twice producing similar results.
Randomization	No randomization was used
Blinding	No blinding was done

Reporting for specific materials, systems and methods

We require information from authors about some types of materials, experimental systems and methods used in many studies. Here, indicate whether each material, system or method listed is relevant to your study. If you are not sure if a list item applies to your research, read the appropriate section before selecting a response.

Materials & experimental systems

n/a	Involved in the study	
☐	☒	Antibodies
☐	☒	Eukaryotic cell lines
☒	☐	Palaeontology
☐	☒	Animals and other organisms
☒	☐	Human research participants
☒	☐	Clinical data

Methods

n/a	Involved in the study	
☒	☐	ChIP-seq
☒	☐	Flow cytometry
☒	☐	MRI-based neuroimaging

Antibodies

Antibodies used	4G2 mAb - Gentry et al. Am J Trop Med Hyg. 1982 May; 31 (3 Pt 1):548-55.<br>4G4 mAb - Clark et al. J. Gen Viril. 2007 Apr; 88(Pt 4):1175-83.
Validation	4G4 antibody was previously validated for ZIKV NS1 detection by immuno-histological staining of mosquitoes in Hugo et al. PLpS Negl Trop Dis. 2019 April; 13(4): e0007281<br>4G2 antibody was previously validated for ZIKV detection by immunofluorescent plaque assay in Setoh et al. Nature Micro. 2019 March; 4(5):876-887.

Eukaryotic cell lines

Policy information about cell lines

Cell line source(s)	Female African green monkey (Cercopithecus aethiops) kidney fibroblasts cells (Vero, ATCC – CCL-81; Vero 76, ATCC – CRL-1587), Aedes aegypti larvae cells Aag2 (ATCC – CCL-125) and Aedes albopictus larvae cells C6/36 (ATCC – CRL-1660) were obtained from the ATCC). Aedes albopictus larvae cells RML-1241 were a generous gift from Prof. Robert Tesh (UTMB, USA).
Authentication	Cell lines were not genetically authenticated, but their morphologies were visually confirmed
Mycoplasma contamination	All cell lines were tested and confirmed to be mycoplasma-free
Commonly misidentified lines (See ICLAC register)	Not used

Animals and other organisms

Policy information about studies involving animals; ARRIVE guidelines recommended for reporting animal research

Laboratory animals	Aedes aegypti mosquitoes. Aedes aegypti mosquitoes were obtained from a colony housed at Public Health Virology, Forensic and Scientific Services (FSS), Brisbane, Australia. All mosquito exposures were conducted in Biological Safety Level (BSL) 3 insectaries at FSS or QIMR Berghofer Medical Research Institute. Aedes aegypti mosquitoes were hatched and reared in plastic trays containing 3L of rainwater at a density of 300 larvae per tray. Larvae were fed ground TetraMin Tropical Fish Food flakes (Tetra, Melle, Germany) ad libitum. Pupae were transferred to a dish of water inside BugDorm cages cage for adult emergence. Adult mosquitoes were provided with cotton wool soaked with 10% sugar solution which was withdrawn 48 hr prior to blood feeding. Three to five day old female mosquitoes were used in experiments.
Wild animals	NA
Field-collected samples	The mosquito colony was established from eggs collected from Innisfail, Australia in April 2017 and the F3-5 generations were used for the experiments.
Ethics oversight	No ethical approval or guidance was required

Note that full information on the approval of the study protocol must also be provided in the manuscript.
