# Supplementary material for: Zika virus noncoding RNA suppresses apoptosis and is required for virus transmission by mosquitoes
Source: Nat Commun. 2020 May 5;11:2205. doi: 10.1038/s41467-020-16086-y (PMC7200751; doi:10.1038/s41467-020-16086-y)
Supplement: Supplementary file 4 — Description of Additional Supplementary Files [file 41467_2020_16086_MOESM4_ESM.pdf]

Description of Additional Supplementary Files

File Name: Supplementary Data 1
Description:

Normalised read counts and statistics outputs for differential gene expression testing.

Supplementary data file table 1. Differentially expressed genes in mosquitoes infected with WT ZIKV comparing to mock infected mosquitoes; gene_ID – Entrez ID, logFC – log_2 fold change, CPM - counts per million, FDR – false discovery rate, WT – wild type ZIKV, Mut – xrRNA1 ZIKV mutant. Counts are presented as log_2 counts per million and normalised to the library sizes.

Supplementary data file table 2. Differentially expressed genes in mosquitoes infected with xrRNA2' mutant ZIKV comparing to mock infected mosquitoes; gene_ID – Entrez ID, logFC – log_2 fold change, CPM - counts per million, FDR – false discovery rate, WT – wild type ZIKV, Mut – xrRNA1 ZIKV mutant. Counts are presented as log_2 counts per million and normalised to the library sizes.

Supplementary data file table 3. Comparison of differentially expressed genes between mosquitoes infected with WT and xrRNA2' relative to mock infected mosquitoes; gene_ID – Entrez ID, logFC – log_2 fold change, CPM - counts per million, FDR – false discovery rate, WT – wild type ZIKV, Mut – xrRNA1 ZIKV mutant. Counts are presented as log_2 counts per million and normalised to the library sizes.
